# Identification of Symptomatic Carotid Artery Plaque: A Three-Item Scale Combined Angiography With Optical Coherence Tomography

**DOI:** 10.3389/fnins.2021.792437

**Published:** 2021-12-10

**Authors:** Qingwen Yang, Hongquan Guo, Xuan Shi, Xiaohui Xu, Mingming Zha, Haodi Cai, Dahong Yang, Feihong Huang, Xiaohao Zhang, Qiushi Lv, Rui Liu, Xinfeng Liu

**Affiliations:** ^1^Department of Neurology, Jinling Hospital, Medical School of Southeast University, Nanjing, China; ^2^Department of Neurology, Jinling Hospital, The First School of Clinical Medicine, Southern Medical University, Nanjing, China; ^3^Department of Neurology, Affiliated Jinling Hospital, Medical School of Nanjing University, Nanjing, China

**Keywords:** optical coherence tomography, symptomatic carotid plaque, plaque surface morphology, vulnerable plaque, angiography

## Abstract

**Introduction:** Symptomatic carotid disease conveys a high risk of recurrent stroke. Plaque morphology and specific plaque characteristics are associated with the risk of stroke. This study aimed to evaluate the detailed plaque features by optical coherence tomography (OCT) and develop a simple scale combining clinical indicators, digital subtraction angiography (DSA), and OCT imaging markers to identify symptomatic carotid plaque.

**Methods:** Carotid plaques from consecutive patients who underwent carotid OCT imaging between June 2017 and June 2021 were evaluated. Clinical characteristics, DSA, and OCT data were compared between the symptomatic and asymptomatic groups. Logistic regression was performed to identify the factors associated with symptomatic carotid plaque and to develop a scale. The area under the receiver operating characteristic curve (AUC) was used to evaluate the performance of the scale.

**Results:** A total of 90 carotid plaques from 90 patients were included (symptomatic 35.6%, asymptomatic 64.4%). Three main factors were found to be associated with symptomatic carotid plaque: high-density lipoprotein cholesterol (HDL-C) <0.925 mmol/L (OR, 4.708; 95% CI, 1.640 to 13.517; *P* = 0.004), irregular plaque (OR, 4.017; 95% CI, 1.250 to 12.910; *P* = 0.020), and white thrombus (OR, 4.594; 95% CI, 1.141 to 18.487; *P* = 0.032). The corresponding score of three items produced a scale with good discrimination (AUC, 0.768; 95% CI, 0.665 to 0.871). The optimal cutoff value of the scale was 1.5 points with 59.4% sensitivity and 84.5% specificity.

**Conclusion:** The three-item scale comprising HDL-C <0.925 mmol/L, angiographical irregular plaque, and white thrombus detected by OCT may provide information to identify symptomatic carotid plaque. Further large-scale studies are required to validate whether the symptomatic carotid plaque scale is clinically valuable in recognizing carotid atherosclerosis in the early stages.

## Introduction

Atherosclerotic carotid stenosis is one of the most common causes of ischemic stroke (Holmstedt et al., 2013; Hurford et al., 2020). The prevalence of symptomatic carotid stenosis increases with age, and it is perceived to convey a higher risk of recurrent stroke than asymptomatic carotid stenosis (Hurford et al., 2020; Krist et al., 2021). Advances in imaging techniques enable us to detect carotid plaque features (Saba et al., 2019). Beyond digital subtraction angiography (DSA), which is regarded as the “gold standard” to diagnose carotid stenosis in most randomized trials, several modalities are most often used to detect carotid plaque, including ultrasound, computed tomography, and magnetic resonance imaging (MRI) (Saba et al., 2019, 2021). However, some important plaque features are generally ignored due to the low resolution of common imaging techniques.

Irregular morphology of the luminal surface based on angiography is considered as a risk feature for stroke (Eliasziw et al., 1994; Homburg et al., 2010). Precise imaging techniques are needed to detect detailed plaque features of irregular carotid stenosis. Optical coherence tomography (OCT) is a relatively novel intravascular imaging modality that utilizes near-infrared light to generate high-resolution (15–20 μm) images of intravascular structures. OCT can provide detailed plaque component characterization *in vivo*, such as lipid content, calcification, fibrous cap thickness (FCT), plaque rupture, and thrombus, and it has been validated by histological controls (Meissner et al., 2006; Habara et al., 2018). A small observational OCT-based study demonstrates that vulnerable plaque features, including thin-cap fibroatheroma (TCFA) with rupture and thrombus are more common in symptomatic patients (Jones et al., 2014). Nevertheless, previous studies that investigate the predictive value of plaque imaging in patients with symptomatic and asymptomatic plaques have no combination of clinical factors with multiple imaging biomarkers (Buskens et al., 2004; Afonso et al., 2015; Park et al., 2019). In addition, few studies use multimodal imaging techniques to detect carotid plaque based on OCT, and published research that applies OCT in carotid artery have relatively small sample sizes (Jones et al., 2014; Shindo et al., 2015).

Our study aimed to evaluate the detailed plaque features by OCT and develop a simple scale combining clinical indicators, plaque surface morphology from DSA, and plaque characteristics from OCT to better identify symptomatic carotid plaque.

## Materials and Methods

### Standard Protocol Approvals, Registrations, and Patient Consents

This was a retrospective, observational study of patients admitted to the Department of Neurology, Jinling Hospital between June 2017 and June 2021. The study protocol was approved by the Ethics Committee of Jinling Hospital. Informed consent was obtained from all enrolled patients before DSA and OCT imaging.

### Study Population

From June 2017 to June 2021, 133 consecutive patients admitted to our hospital underwent the OCT examination when carotid artery stenosis was confirmed ≥30% by DSA. Ten patients who underwent OCT examination for evaluation of in-stent restenosis of carotid artery stenting were excluded. Two patients of non-atherosclerotic etiologies were also excluded. Of 125 cervical internal carotid artery lesions in 121 patients, 31 lesions with poor image quality or incomplete target lesions imaging were excluded. We selected the side with a severe degree of stenosis if patients had both sides of lesions. Finally, 90 lesions from 90 patients were enrolled in our study.

### Data Collection

Demographics, clinical characteristics, and laboratory examination were reviewed. Demographic data included age and gender. Clinical data, including the history of hypertension, diabetes mellitus, hyperlipidemia, coronary heart disease, current smoking, previous stroke, usage of antiplatelet and statin drugs, degree of carotid stenosis, and stenting, were collected. Stroke risk factors were defined according to current guidelines (Kernan et al., 2014). Lipid levels were tested by standard enzymatic techniques, including total cholesterol (TC), triglyceride (TG), high-density lipoprotein cholesterol (HDL-C), and low-density lipoprotein cholesterol (LDL-C). We separated the patients into symptomatic and asymptomatic groups according to the presence or absence of previous symptoms. Symptomatic patients were defined as those who had a cerebral ischemic event, including ischemic stroke or transient ischemic attack, in the corresponding vascular territory within 6 months before the hospitalization (Jones et al., 2014).

### Digital Subtraction Angiography Procedure and Optical Coherence Tomography Image Protocol

A DynaCT angiography scanner (Siemens Axiom Artis dTA, Siemens Healthcare, Erlangen, Germany) was used for the DSA examination. The degree of carotid stenosis was evaluated according to North America Symptomatic Carotid Endarterectomy Trial (NASCET) criteria (Barnett et al., 1998). Frequency domain OCT systems (ILUMEN OPTIS System or C7-XR, St. Jude Medical, Abbott Vascular, United States) and 2.7-F OCT imaging catheters (C7 Dragonfly Catheter or Dragonfly Duo Catheter, St. Jude Medical, Abbott Vascular, United States) were used for OCT evaluation. The OCT catheter was inserted through an 8-F sheath over the 0.014-inch guidewire of the filter, and advanced distal to the ICA lesion. OCT image was acquired by the injection of 20 mL undiluted iodixanol 320 (GE Healthcare Ireland Limited, County Cork, Ireland) through the guiding catheter at the flow rate of 10 mL/s. Images were calibrated by adjustment of the Z-offset. Automatic pullbacks covered 54 mm of the vessel at a velocity of 20 mm/s or 25 mm/s (Liu et al., 2015). OCT images were stored and analyzed subsequently using proprietary software (ILUMEN OPTIS System, St. Jude Medical, Abbott Vascular, United States). The DSA and OCT procedures were performed by interventional neurologists with extensive experience in carotid OCT.

### Angiographic Plaque Surface Morphology and Optical Coherence Tomography Image Analysis

Both DSA and OCT images were analyzed by two independent investigators (QWY and HQG) who were blinded to the clinical details. When disparities arose regarding the evaluation results, the consensus was achieved with the assistance of a third independent investigator (XS). To determine the reproducibility of DSA and OCT image assessment, data were analyzed repeatedly 1 month after initial analysis by the same investigator (QWY).

Carotid plaque surface morphology was classified as irregular and smooth according to the DSA. Plaques were defined as irregular if pre- or poststenotic dilatation was present and/or if the plaque surface morphology showed irregularities (de Weert et al., 2009). Plaques were defined as smooth if there was no major surface irregularity visible on angiographical images (Rothwell et al., 2000b). Of the irregular plaques, plaques were further categorized as non-ulcerated and ulcerated plaque. Plaques were defined as ulcerated if the extension of contrast material was present beyond the vascular lumen into the surrounding plaque (de Weert et al., 2009). The ulcer was reserved for cavities measuring at least 2 mm (Eyding et al., 2011).

Plaque features were evaluated based on previously published consensus standards for OCT (Tearney et al., 2012). A lesion was identified as a mass lesion within the artery wall; focal intimal thickening; or loss of the layered intima, media, adventitia architecture. Lipid plaque was defined as a diffusely bordered signal-poor region with signal attenuation within a lesion that is covered by a signal-rich layer and lipid-rich plaque as a plaque with lipid core >90°. FCT was measured at the thinnest part three times, and the average value was calculated. TCFA was defined as a plaque with a maximal lipid arc greater than 90° and the thinnest FCT less than or equal to 65 μm. Cholesterol crystal was defined as a thin, linear region of high intensity, usually existing beside the lipid core. Macrophage accumulation appeared as signal-rich, distinct, or confluent punctate regions that exceed the intensity of background speckle noise. Neovascularization was defined as signal-absent holes within a plaque measuring between 50 and 300 μm in diameter and visible on at least three consecutive frames on pullback imaging (Tearney et al., 2012). Plaque rupture was defined as the presence of fibrous cap discontinuity with a clear cavity formed inside the plaque (Kubo et al., 2007). Calcification was identified as the presence of a signal-poor region with a well-delineated border. Spotty calcification was defined as the presence of lesions <4 mm in length and containing an arc of calcification ≤90° (Kataoka et al., 2014). Large calcification was defined as calcification with an arc of >90° (Mizukoshi et al., 2013). Ruptured calcified nodule was defined as an irregular, protruding calcification with irregular luminal surface and disrupted fibrous cap (Jia et al., 2013). Intraluminal thrombus appeared as a mass attached to the surface of the vessel wall or floating within the vessel lumen. Red thrombus was recognized as high backscattering and attenuation. White thrombus was recognized as homogeneous backscattering and low attenuation (Tearney et al., 2012). A complicated American Heart Association (AHA-VI) type plaque was evaluated as having at least one of the following three features present: ruptured TCFA, intraluminal thrombus, and ruptured calcified nodule (Jones et al., 2014).

### Statistical Analysis

Categorical variables were expressed as numbers (percentages). Continuous variables were displayed as mean ± standard deviation (SD) or medians (interquartile range). Univariate analysis was performed between the symptomatic and asymptomatic groups by using *t*-test, Mann-Whitney *U* test, Chi-square test, or Fisher exact test. Inter- and intraobserver agreements were determined using the Cohen Kappa test for categorical variables and intra-class correlation test for continuous variables. Variables with a *P*-value <0.05 on univariate analysis were included in multiple logistic regression. Continuous variables were converted into dichotomous variables before enrolling in the regression model, and the receiver operating characteristic (ROC) curve was used to calculate the cutoff value by maximizing the Youden index (sensitivity + specificity − 1). Variables were selected after checking for multicollinearity using the variance inflation factor (VIF). Logistic regression analysis (forward stepwise) was taken to generate the regression model on the dichotomous symptomatic carotid plaque.

Overall performance of the model was evaluated by the area under the ROC curve (AUC). β-coefficients from the regression model were used to generate the scoring system of the symptomatic scale. The optimal cutoff value of the scale was calculated using the ROC curve analysis.

A two-sided *P*-value <0.05 was considered statistically significant. Statistical analysis was conducted using SPSS 25.0 (IBM Corp., Armonk, NY, United States) and R statistical software, version 4.1.0.

## Results

### Patient Characteristics in Symptomatic and Asymptomatic Group

Of all 90 patients included in the study, the mean age was 67.0 years old, and 70 patients (77.8%) were male. Thirty-two patients (35.6%) were categorized as the symptomatic group and 58 patients (64.4%) as the asymptomatic group. Twenty-four patients (75.0%) underwent stenting of targeted carotid, contralateral carotid, or posterior circulation in the symptomatic group, whereas 39 patients (67.2%) underwent it in the asymptomatic group. Detailed demographic and clinical data are shown in Table 1. Symptomatic patients had lower levels of HDL-C (0.9 mmol/L [0.8–1.0] vs. 1.0 mmol/L [0.9–1.1], *P* = 0.040). There was no significant difference in other clinical characteristics between symptomatic and asymptomatic patients (*P* > 0.05).

**TABLE 1:** Demographic and clinical data of the study population.

Variable	Symptomatic (*n* = 32)	Asymptomatic (*n* = 58)	*P-*value
Age, years	67.0 (61.2–71.8)	66.5 (60.8–69.2)	0.396
Male	27 (84.4)	43 (74.1)	0.263
**Clinical features**			
Hypertension	26 (81.3)	47 (81.0)	0.980
Diabetes mellitus	9 (28.1)	28 (48.3)	0.063
Hyperlipidemia	5 (15.6)	9 (15.5)	0.989
Coronary heart disease	10 (31.3)	15 (25.9)	0.585
Current smoking	15 (46.9)	25 (43.1)	0.730
Previous stroke	13 (40.6)	15 (25.9)	0.148
**Current medications**			
Aspirin	17 (53.1)	24 (41.4)	0.284
Clopidogrel	10 (31.3)	11 (19.0)	0.187
Statin	15 (46.9)	22 (37.9)	0.409
**Serum lipid, mmol/L**			
Total cholesterol	3.4 (2.8–3.9)	3.5 (2.9–4.1)	0.423
Triglycerides	1.3 (0.9–1.9)	1.2 (1.0–1.8)	0.723
HDL-C	0.9 (0.8–1.0)	1.0 (0.9–1.1)	0.040
LDL-C	1.9 (1.5–2.3)	1.8 (1.5–2.4)	0.960
Carotid stenosis ≥50%	28 (87.5)	50 (86.2)	0.863
Stenting	24 (75.0)	39 (67.2)	0.442

*Data were expressed as median (interquartile range) or number (percentage) as appropriate. HDL-C, high density lipoprotein-cholesterol; LDL-C, low density lipoprotein-cholesterol.*

### Comparison of Carotid Plaque Characteristics Between Symptomatic and Asymptomatic Group

**FIGURE 1:**
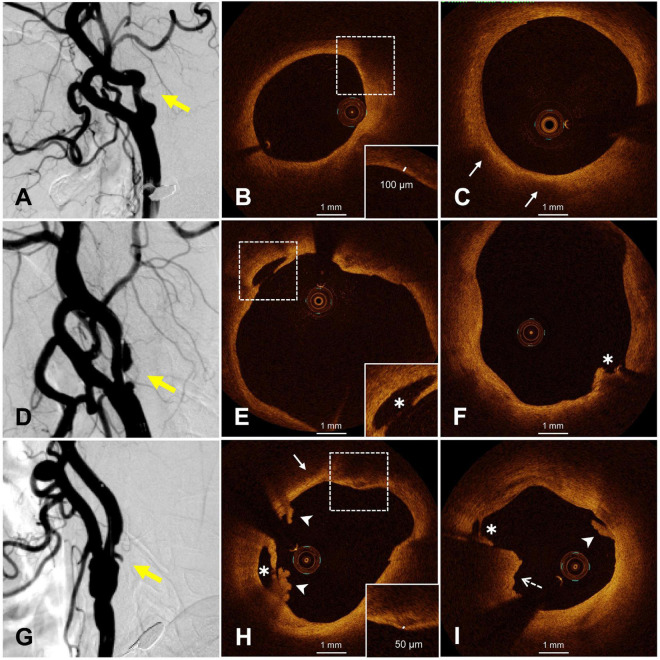
Representative OCT images of smooth and irregular plaques. **(A)** Smooth plaque (yellow arrow), **(B)** fibrous cap thickness >65 μm (dotted frame), **(C)** lipid pool (white arrow), **(D,G)** irregular plaques, **(E,F)** plaque rupture (white asterisk), **(H)** white thrombus (white arrowhead), rupture (white asterisk), thin fibrous cap (dotted frame), lipid pool (white arrow), **(I)** white thrombus (white arrow), red thrombus (dotted arrow), and rupture (white asterisk). OCT, optical coherence tomography.

Representative OCT and DSA images of carotid plaques can be seen in Figure 1. Comparison of carotid plaque characteristics between the two groups are summarized in Table 2. Plaque surface morphology of symptomatic patients was more likely to be irregular (81.3% vs. 53.4%, *P* = 0.009) and ulcerated (46.9% vs. 22.4%, *P* = 0.016). Compared with asymptomatic patients, plaque of symptomatic patients was more prone to be ruptured (68.8% vs. 43.1%, *P* = 0.020). AHA type VI plaque was observed more frequently in symptomatic patients (65.6% vs. 37.9%, *P* = 0.012). These were largely due to the higher incidence of ruptured TCFA (59.4% vs. 34.5%, *P* = 0.023) and intraluminal thrombus (37.5% vs. 13.8%, *P* = 0.010). Symptomatic patients demonstrated higher rates of white thrombus (28.1% vs. 6.9%, *P* = 0.015), and red thrombus was numerically more prevalent in symptomatic patients (25.0% vs. 8.6%, *P* = 0.071). No significant difference was found in other plaque characteristics (*P* > 0.05).

**TABLE 2:** Plaque characteristics in symptomatic and asymptomatic group.

Variable	Symptomatic (*n* = 32)	Asymptomatic (*n* = 58)	*P-*value
**DSA**			
Stenosis severity			0.487
30–49%	4 (12.5)	8 (13.8)	
50–69%	6 (18.8)	17 (29.3)	
70–99%	22 (68.8)	33 (56.9)	
Irregular	26 (81.3)	31 (53.4)	0.009
Ulcerated	15 (46.9)	13 (22.4)	0.016
**OCT**			
Lipid-rich	24 (75.0)	40 (69.0)	0.545
FCT, μm	44.5 (34.4–61.1)	53.6 (40.0–78.9)	0.058
TCFA	21 (65.6)	31 (53.4)	0.263
Rupture	22 (68.8)	25 (43.1)	0.020
Calcification	22 (68.8)	44 (75.9)	0.465
Spotty calcification	22 (68.8)	44 (75.9)	0.465
Large calcification	2 (6.3)	9 (15.5)	0.199
Cholesterol crystals	16 (50.0)	23 (39.7)	0.343
Macrophage accumulation	14 (43.8)	28 (48.3)	0.680
Neovascularization	9 (28.1)	14 (24.1)	0.678
Ruptured TCFA	19 (59.4)	20 (34.5)	0.023
Ruptured calcified nodule	3 (9.4)	3 (5.2)	0.746
Intraluminal thrombus	12 (37.5)	8 (13.8)	0.010
Red thrombus	8 (25.0)	5 (8.6)	0.071
White thrombus	9 (28.1)	4 (6.9)	0.015
AHA type VI plaque	21 (65.6)	22 (37.9)	0.012

*Data were expressed as median (interquartile range) or number (percentage) as appropriate. DSA, digital subtraction angiography; OCT, optical coherence tomography; FCT, fibrous cap thickness; TCFA, thin-cap fibroatheroma; AHA, American Heart Association.*

The evaluation of plaque morphology by DSA showed good interobserver reproducibility (*k* = 0.94). Interobserver agreement between two investigators for the identification of plaque qualitative characteristics by OCT was all very good with kappa coefficient >0.80. The measurement of the FCT also showed good interobserver reproducibility (intraclass correlation coefficient = 0.803). Intraobserver agreement, evaluated twice by one observer within 1 month, showed good reproducibility (qualitative indicators, *k* > 0.80; FCT, intraclass correlation coefficient = 0.853).

### The Scale Combining Clinical and Imaging Indicators to Identify Symptomatic Carotid Plaque

**FIGURE 2:**
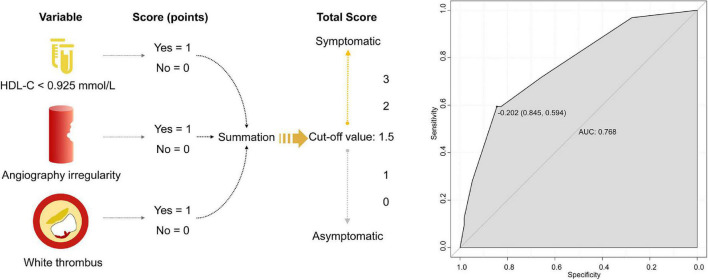
The three-item scale of symptomatic carotid plaque and the ROC curve. HDL-C, high-density lipoprotein cholesterol; ROC, receiver operating characteristic; AUC, operating characteristic curve.

After excluding collinearity and enrolling all potential predictors into the logistic regression, we found that HDL-C <0.925 mmol/L (OR, 4.708; 95% CI, 1.640 to 13.517; *P* = 0.004), irregular plaque (OR, 4.017; 95% CI, 1.250 to 12.910; *P* = 0.020), and white thrombus (OR, 4.594; 95% CI, 1.141 to 18.487; *P* = 0.032) were associated with symptomatic carotid plaque (Table 3). The β-coefficients of the three variables were approximate to 1:1:1, and each indicator was assigned a score of 1 (Figure 2 and Table 3). The illustration of the symptomatic carotid plaque scale is shown in Figure 2. Our three-item scale comprises a clinical indicator, the plaque surface morphology from DSA, and a specific plaque feature from OCT: HDL-C <0.925 mmol/L, irregular plaque, and white thrombus. The model had good discrimination (AUC, 0.768; 95% CI, 0.665 to 0.871). The optimal cutoff value of the scale was 1.5 points with 59.4% sensitivity and 84.5% specificity (Figure 2).

**TABLE 3:** Clinical and imaging indicators to identify symptomatic carotid plaque in the final multivariable regression model.

Variable	β-coefficient	Standard error	Wald	OR (95% CI)	*P-*value
**Clinical indicator**					
HDL-C <0.925 mmol/L	1.549	0.538	8.291	4.708 (1.640–13.517)	0.004
**DSA indicator**					
Irregular	1.391	0.596	5.449	4.017 (1.250–12.910)	0.020
**OCT indicator**					
White thrombus	1.524	0.711	4.601	4.592 (1.141–18.487)	0.032

*The final multivariable regression model was adjusted for diabetes mellitus. DSA, digital subtraction angiography; OCT, optical coherence tomography; OR, odds ratio; CI, confidence interval.*

## Discussion

We describe a simple scale, including clinical indicators, angiographical plaque surface morphology, and plaque characteristics assessed by OCT in patients with carotid artery stenosis. The use of multimodal imaging techniques comprising the traditional imaging method and a new high-resolution imaging method to detect carotid plaque help to comprehensively analyze the risk of plaque. Our study indicates the three-item scale consisting of HDL-C <0.925 mmol/L, irregular plaque, and white thrombus has a promising value to identify clinical presentations of carotid plaque.

Multiple biomarkers and subsequent models have been developed to assess the risk of stroke in patients with carotid stenosis (Marnane et al., 2014; Naylor et al., 2015). Clinical risk factors (male gender, age >75 years, and recent symptomatic status, etc.) and imaging parameters (a large plaque area, irregular stenosis, increasing stenosis severity, a low gray-scale median value, intra-plaque hemorrhage at MRI, etc.) have been used to predict the carotid artery-related risk of early or late stroke (Naylor et al., 2015; Fabiani et al., 2020). Ultrasound, computed tomography angiography, and magnetic resonance angiography are widespread imaging methods to screen carotid stenosis but have limited accuracy in recognizing plaque morphology (Saxena et al., 2019; Mushenkova et al., 2020). More precise imaging techniques are needed to distinguish plaque components and structures.

Optical coherence tomography is called “optical biopsy *in vivo*” for its super high-resolution arterial wall imaging, which can detect detailed plaque components *in vivo* with a good agreement with histology (Habara et al., 2018; Mushenkova et al., 2020). This novel imaging technique is also frequently used in the identification of plaque vulnerability and evaluation of stent–vessel interactions (Jones et al., 2014; Liu et al., 2015; Shi et al., 2020). In our study, white thrombus, a type of platelet-rich thrombus, was identified to be the imaging indicator from OCT to recognize symptomatic carotid plaque. Thrombus is an important component of complicated AHA-VI type plaque (Stary et al., 1995). In cerebral large vessel occlusion, high platelet content of thrombus is associated with a large artery atherosclerosis source (34). There is also evidence that intraluminal thrombus is identified as the independent predictor of symptomatic patients, which is similar to our findings (Jones et al., 2014). In other carotid stenosis studies, special plaque features, such as plaque rupture and thin fibrous cap, are also associated with an increased risk of stroke (Takaya et al., 2006; Sadat et al., 2009).

Increasing clinical evidence suggests that plaque morphologic features and plaque vulnerability have relevance to the risk of stroke (Rundek, 2007; Saba et al., 2019). Angiographic plaque irregularity is a strong independent predictor of ischemic stroke (Eliasziw et al., 1994; Liapis and Paraskevas, 2006). Carotid plaque surface irregularity, especially plaque ulceration, is reported to be associated with the risk of stroke (Rothwell et al., 2000a; Prabhakaran et al., 2006; Rundek, 2007). In our study, irregular plaque morphology is recognized as the indicator to identify symptomatic carotid plaque rather than ulcerated plaque morphology, which is possibly attributed to the limited number of ulcerated plaques.

Blood cholesterol is an accepted causal risk factor for ischemic vascular disease (Lewington et al., 2007). In observational epidemiological studies, HDL-C concentration is consistently associated inversely with the risk of atherosclerotic events (Libby et al., 2019). In our research, a baseline HDL-C level <0.925 mmol/L was the independent indicator of symptomatic carotid plaque. Prospective observational studies about ischemic heart disease (IHD) have a similar discovery: there is a strong inverse association between HDL-C and risk of IHD, and the association remains after the adjustment for other lipid measures (Lewington et al., 2007; Di Angelantonio et al., 2009). Other lipid measures, including low-density lipoprotein, triglycerides, and lipoprotein (a) are also proved to be associated with atherosclerotic risk, which emphasizes the importance of the regulation of blood lipids (Herrington et al., 2016).

This study has several strengths, including the imaging data collection based on the high-resolution OCT technique for the evaluation of detailed plaque structure and supplemented by the gold standard DSA for the assessment of plaque surface morphology. The combination of two different modalities with complementary strengths allows a more specific and comprehensive evaluation of plaque features. In addition, we included patients with DSA-demonstrated non-stenotic atheromatous disease (stenosis <50%), which provides information for the clinical and imaging characteristics of the symptomatic non-stenotic carotid disease. Given the potential role of non-stenotic carotid plaques in stroke etiology, further research should aim to identify features that predict the risk of non-stenotic carotid plaques becoming symptomatic.

There are also some potential limitations to our study. First, it had a retrospective design with a small sample size in a single center. Second, the scale was based solely on current data, and we did not perform validation in other independent cohorts due to the limited sample. Further research is needed to use the scale to identify symptomatic carotid plaque. Third, the long-term follow-up data after the intervention was not reported. Recurrence of stroke in patients with symptomatic carotid plaque and the first occurrence of stroke in asymptomatic patients will be analyzed in our subsequent research. Lesion evolution and imaging evaluation after carotid artery stenting will also be focused on in further studies.

## Conclusion

The three-item scale comprising dichotomous HDL-C, irregular plaque, and white thrombus may provide information to identify symptomatic carotid plaque. The scale may help in recognizing carotid atherosclerosis diseases in the early stages and contribute to making clinical decisions on the follow-up treatment profile. Future studies in other independent cohorts are needed to validate the clinical value of the symptomatic carotid plaque scale.

## Data Availability Statement

The raw data supporting the conclusions of this article will be made available by the authors, without undue reservation.

## Ethics Statement

The studies involving human participants were reviewed and approved by Ethics Committee of Jinling Hospital. The patients/participants provided their written informed consent to participate in this study. Written informed consent was obtained from the individual(s) for the publication of any potentially identifiable images or data included in this article.

## Author Contributions

QY, HG, RL, and XL: study design. QY, HG, XS, XX, and FH: data collection. QY, HG, and RL: analysis and interpretation of data. QY and HG: writing of the manuscript. XS, XX, MZ, HC, DY, XZ, QL, RL, and XL: revision of the manuscript. All authors contributed to the article and approved the submitted version.

## Conflict of Interest

The authors declare that the research was conducted in the absence of any commercial or financial relationships that could be construed as a potential conflict of interest.

## Publisher’s Note

All claims expressed in this article are solely those of the authors and do not necessarily represent those of their affiliated organizations, or those of the publisher, the editors and the reviewers. Any product that may be evaluated in this article, or claim that may be made by its manufacturer, is not guaranteed or endorsed by the publisher.
